# 2′-Methyl-2′-nitro-1′-phenyl-2′,3′,5′,6′,7′,7a’-hexa­hydro­spiro­[indoline-3,3′-1′*H*-pyrrolizin]-2-one

**DOI:** 10.1107/S1600536808020837

**Published:** 2008-07-16

**Authors:** Yaghoub Sarrafi, Kamal Alimohammadi

**Affiliations:** a Department of Chemistry, University of Mazandaran, 47415 Babolsar, Iran

## Abstract

The title compound, C_21_H_21_N_3_O_3_, was synthesized by a multi-component 1,3-dipolar cyclo­addition of azomethine ylide, derived from isatin and proline by a deca­rboxylative route, and (*E*)-1-phenyl-2-nitro­propene. In the mol­ecule, the spiro junction links a planar oxindole ring and a pyrrolidine ring in an envelope conformation. The mol­ecular packing is stabilized by an inter­molecular N—H⋯N inter­action of the oxindole and pyrrolizidine rings.

## Related literature

For related literature, see: Daly *et al.* (1986 ▶); Grigg & Sridharan (1993 ▶); Padwa (1984 ▶); Usha, Selvanayagam, Velmurugan, Ravikumar & Poornachandran (2005 ▶); Usha, Selvanayagam, Velmurugan, Ravikumar & Raghunathan (2005 ▶); Waldmann (1995 ▶).
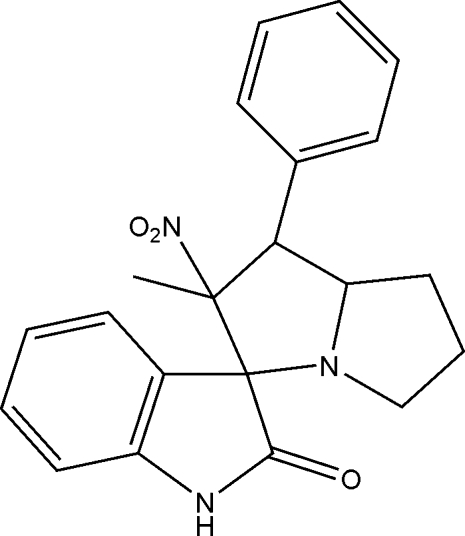


## Experimental

### 

#### Crystal data

C_21_H_21_N_3_O_3_
*M* *_r_* = 363.41
Monoclinic, 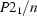

*a* = 7.8524 (16) Å
*b* = 25.656 (6) Å
*c* = 9.1767 (19) Å
β = 110.489 (4)°
*V* = 1731.8 (6) Å^3^
*Z* = 4
Mo *K*α radiation
μ = 0.10 mm^−1^
*T* = 120 (2) K
0.21 × 0.18 × 0.15 mm

#### Data collection

Bruker SMART 1000 CCD area-detector diffractometer
Absorption correction: multi-scan (*SADABS*; Sheldrick, 1996 ▶) *T*_min_ = 0.980, *T*_max_ = 0.989
16064 measured reflections
3773 independent reflections
2183 reflections with *I* > 2σ(*I*)
*R*_int_ = 0.064

#### Refinement

*R*[*F* ^2^ > 2σ(*F* ^2^)] = 0.051
*wR*(*F* ^2^) = 0.102
*S* = 1.01
3773 reflections
245 parameters
H-atom parameters constrained
Δρ_max_ = 0.25 e Å^−3^
Δρ_min_ = −0.28 e Å^−3^

### 

Data collection: *SMART* (Bruker, 2007 ▶); cell refinement: *SAINT* (Bruker, 2007 ▶); data reduction: *SAINT*; program(s) used to solve structure: *SHELXTL* (Sheldrick, 2008 ▶); program(s) used to refine structure: *SHELXTL*; molecular graphics: *SHELXTL*; software used to prepare material for publication: *SHELXTL*.

## Supplementary Material

Crystal structure: contains datablocks I, global. DOI: 10.1107/S1600536808020837/om2246sup1.cif

Structure factors: contains datablocks I. DOI: 10.1107/S1600536808020837/om2246Isup2.hkl

Additional supplementary materials:  crystallographic information; 3D view; checkCIF report

## Figures and Tables

**Table 1:** Hydrogen-bond geometry (Å, °)

*D*—H⋯*A*	*D*—H	H⋯*A*	*D*⋯*A*	*D*—H⋯*A*
N1′—H1′⋯N1^i^	0.85	2.21	2.992 (3)	151

Symmetry code: (i) 
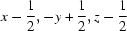
.

**Fig. 1.**
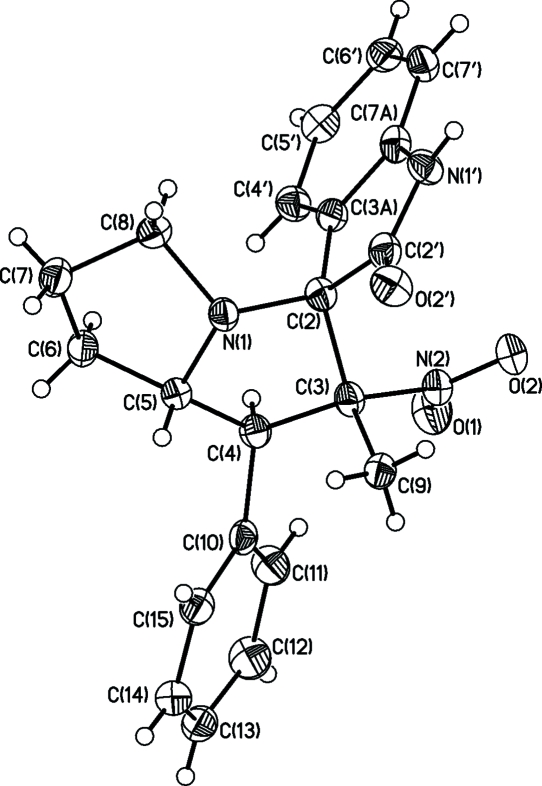
The molecular structure of the title compound with the numbering scheme for the atoms and 50% probability displacement ellipsoids.

**Fig. 2.**
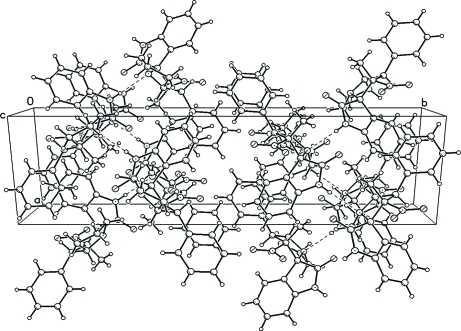
Packing diagram of the molecules, viewed down the c axis.

## supplementary crystallographic information

### Comment

Multicomponent 1,3-dipolar cycloaddition reactions are considered to be one of the most useful processes for the construction of five-membered heterocyclic ring systems (Padwa, 1984; Grigg & Sridharan, 1993). These strategies offer significant advantages over more traditional approaches, allowing the construction of complex molecular architectures from easily available starting materials in a single synthetic operation without the need for isolation of intermediates. Particularly, the chemistry of the azomethine ylide has gained significance in recent years for the construction of nitrogen containing five-membered heterocycles, which are often the central ring systems of numerous natural products (Daly *et al.*, 1986; Waldmann, 1995). In contrast to similar compounds (Usha, Selvanayagam, Velmurugan, Ravikumar & Poornachandran, 2005; Usha, Selvanayagam, Velmurugan, Ravikumar & Raghunathan, 2005); Waldmann (1995)), in which the carbon atom bearing nitro group is bonded to the pyrrolidine ring, in the title compound it is bonded to the oxindole ring (Fig. 1). In the crystal structure, *N*—H···H hydrogen bonds link neighboring molecules. Molecules (Fig. 2) are also stacked in a side by side fashion along the *c* axis through π···π interaction and are further linked by a few intermolecular C—H···π interactions,

### Refinement

The hydrogen atom of the NH group was found in difference Fourier synthesis. The H(C) atom positions were calculated. H(N) atom was refined in isotropic approximation in riding model, the H(C) atoms were refined in isotropic approximation in riding model with with the *U*_iso_(H) parameters equal to 1.2 *U*_eq_(Ni), 1.2 *U*_eq_(Ci) or 1.5 *U*_eq_(Cii), where U(Ci) and U(Cii) are respectively the equivalent thermal parameters of the (CH or CH_2_) and CH_3_ carbon atoms to which the corresponding H atoms are bonded.

### Crystal data

**Table:**

C_21_H_21_N_3_O_3_	*F*_000_ = 768
*M**_r_* = 363.41	*D*_x_ = 1.394 Mg m^−^^3^
Monoclinic, *P*2_1_/*n*	Mo *K*α radiation λ = 0.71073 Å
Hall symbol: -P 2yn	Cell parameters from 792 reflections
*a* = 7.8524 (16) Å	θ = 3–23º
*b* = 25.656 (6) Å	µ = 0.10 mm^−^^1^
*c* = 9.1767 (19) Å	*T* = 120 (2) K
β = 110.489 (4)º	Prism, colorless
*V* = 1731.8 (6) Å^3^	0.21 × 0.18 × 0.15 mm
*Z* = 4	

### Data collection

**Table:**

Bruker SMART 1000 CCD area-detector diffractometer	3773 independent reflections
Radiation source: fine-focus sealed tube	2183 reflections with *I* > 2σ(*I*)
Monochromator: graphite	*R*_int_ = 0.064
*T* = 120(2) K	θ_max_ = 27.0º
φ and ω scans	θ_min_ = 1.6º
Absorption correction: multi-scan(SADABS; Sheldrick, 1996)	*h* = −10→10
*T*_min_ = 0.980, *T*_max_ = 0.989	*k* = −32→32
16064 measured reflections	*l* = −11→11

### Refinement

**Table:**

Refinement on *F*^2^	Secondary atom site location: difference Fourier map
Least-squares matrix: full	Hydrogen site location: inferred from neighbouring sites
*R*[*F*^2^ > 2σ(*F*^2^)] = 0.051	H-atom parameters constrained
*wR*(*F*^2^) = 0.103	*w* = 1/[σ^2^(*F*_o_^2^) + (0.01*P*)^2^ + 1.6*P*] where *P* = (*F*_o_^2^ + 2*F*_c_^2^)/3
*S* = 1.01	(Δ/σ)_max_ < 0.001
3773 reflections	Δρ_max_ = 0.25 e Å^−^^3^
245 parameters	Δρ_min_ = −0.27 e Å^−^^3^
Primary atom site location: structure-invariant direct methods	Extinction correction: none

### Special details

**Table:**

Experimental. A mixture of isatin (0.147 g, 1 mmol), proline (0.115 g, 1 mmol), and (*E*)-1-phenyl-2-nitropropene (0.163 g, 1 mmol) in ethanol (10 ml) was stirred at reflux for 1 h. After completion of the reaction, as indicated by TLC, to the solution was added water (25 ml), and the precipitated solid was separated by filtration. The pure cycloadduct was obtained by recrystallization from ethanol.
Geometry. All e.s.d.'s (except the e.s.d. in the dihedral angle between two l.s. planes) are estimated using the full covariance matrix. The cell e.s.d.'s are taken into account individually in the estimation of e.s.d.'s in distances, angles and torsion angles; correlations between e.s.d.'s in cell parameters are only used when they are defined by crystal symmetry. An approximate (isotropic) treatment of cell e.s.d.'s is used for estimating e.s.d.'s involving l.s. planes.
Refinement. Refinement of *F*^2^ against ALL reflections. The weighted *R*-factor *wR* and goodness of fit *S* are based on *F*^2^, conventional *R*-factors *R* are based on *F*, with *F* set to zero for negative *F*^2^. The threshold expression of *F*^2^ > σ(*F*^2^) is used only for calculating *R*-factors(gt) *etc*. and is not relevant to the choice of reflections for refinement. *R*-factors based on *F*^2^ are statistically about twice as large as those based on *F*, and *R*- factors based on ALL data will be even larger.

### Fractional atomic coordinates and isotropic or equivalent isotropic displacement parameters (Å^2^)

**Table:**

	*x*	*y*	*z*	*U*_iso_*/*U*_eq_	
N1	0.1573 (3)	0.19274 (7)	0.7966 (2)	0.0215 (4)	
C2	0.1056 (3)	0.17387 (9)	0.6367 (3)	0.0210 (5)	
C3	0.2848 (3)	0.14420 (9)	0.6478 (3)	0.0218 (5)	
C4	0.3236 (3)	0.11281 (9)	0.8003 (3)	0.0209 (5)	
H4A	0.2295	0.0859	0.7796	0.025*	
C5	0.2810 (3)	0.15438 (9)	0.9046 (3)	0.0212 (5)	
H5A	0.3944	0.1722	0.9645	0.025*	
C6	0.1843 (3)	0.13826 (9)	1.0155 (3)	0.0250 (6)	
H6A	0.2702	0.1284	1.1166	0.030*	
H6B	0.1005	0.1097	0.9735	0.030*	
C7	0.0836 (3)	0.18844 (9)	1.0247 (3)	0.0273 (6)	
H7A	−0.0140	0.1817	1.0640	0.033*	
H7B	0.1657	0.2141	1.0906	0.033*	
C8	0.0098 (3)	0.20643 (10)	0.8562 (3)	0.0250 (6)	
H8A	−0.1020	0.1883	0.7986	0.030*	
H8B	−0.0129	0.2437	0.8495	0.030*	
C9	0.4441 (3)	0.17830 (9)	0.6496 (3)	0.0245 (6)	
H9A	0.5437	0.1566	0.6496	0.037*	
H9B	0.4812	0.1996	0.7413	0.037*	
H9C	0.4079	0.2002	0.5589	0.037*	
C10	0.5061 (3)	0.08576 (9)	0.8734 (3)	0.0208 (5)	
C11	0.5235 (3)	0.03378 (10)	0.8389 (3)	0.0285 (6)	
H11A	0.4285	0.0173	0.7622	0.034*	
C12	0.6799 (4)	0.00634 (10)	0.9170 (3)	0.0333 (6)	
H12A	0.6905	−0.0282	0.8902	0.040*	
C13	0.8199 (3)	0.02934 (10)	1.0338 (3)	0.0298 (6)	
H13A	0.9226	0.0101	1.0894	0.036*	
C14	0.8070 (3)	0.08119 (10)	1.0682 (3)	0.0280 (6)	
H14A	0.9018	0.0972	1.1465	0.034*	
C15	0.6529 (3)	0.10927 (10)	0.9861 (3)	0.0254 (6)	
H15A	0.6475	0.1446	1.0068	0.030*	
N2	0.2395 (3)	0.11023 (8)	0.5039 (2)	0.0241 (5)	
O1	0.2495 (2)	0.06314 (7)	0.5158 (2)	0.0346 (4)	
O2	0.1929 (2)	0.13377 (7)	0.37859 (19)	0.0310 (4)	
N1'	−0.1068 (3)	0.21391 (8)	0.4167 (2)	0.0247 (5)	
H1'	−0.1647	0.2377	0.3535	0.030*	
C2'	0.0582 (3)	0.22165 (9)	0.5263 (3)	0.0245 (5)	
O2'	0.1511 (2)	0.26105 (6)	0.54370 (19)	0.0298 (4)	
C3A	−0.0662 (3)	0.14082 (9)	0.5689 (3)	0.0215 (5)	
C4'	−0.1202 (3)	0.09354 (9)	0.6109 (3)	0.0248 (6)	
H4D	−0.0455	0.0761	0.6989	0.030*	
C5'	−0.2865 (3)	0.07228 (10)	0.5209 (3)	0.0271 (6)	
H5D	−0.3224	0.0403	0.5482	0.032*	
C6'	−0.3983 (3)	0.09824 (10)	0.3915 (3)	0.0266 (6)	
H6D	−0.5086	0.0833	0.3316	0.032*	
C7'	−0.3494 (3)	0.14616 (10)	0.3490 (3)	0.0252 (6)	
H7D	−0.4259	0.1641	0.2628	0.030*	
C7A	−0.1828 (3)	0.16639 (9)	0.4391 (3)	0.0231 (5)	

### Atomic displacement parameters (Å^2^)

**Table:**

	*U*^11^	*U*^22^	*U*^33^	*U*^12^	*U*^13^	*U*^23^
N1	0.0226 (11)	0.0248 (11)	0.0180 (10)	0.0022 (9)	0.0084 (9)	−0.0003 (8)
C2	0.0219 (13)	0.0213 (13)	0.0192 (12)	0.0010 (10)	0.0066 (10)	−0.0011 (10)
C3	0.0227 (13)	0.0227 (13)	0.0187 (12)	−0.0006 (10)	0.0058 (10)	−0.0016 (10)
C4	0.0227 (13)	0.0201 (12)	0.0196 (12)	−0.0022 (10)	0.0072 (10)	−0.0008 (10)
C5	0.0206 (12)	0.0239 (13)	0.0191 (12)	0.0012 (10)	0.0069 (10)	−0.0007 (10)
C6	0.0292 (14)	0.0265 (14)	0.0219 (13)	0.0027 (11)	0.0122 (11)	0.0021 (11)
C7	0.0309 (14)	0.0290 (14)	0.0252 (14)	0.0025 (11)	0.0136 (12)	0.0001 (11)
C8	0.0219 (13)	0.0284 (14)	0.0257 (13)	0.0032 (11)	0.0098 (11)	−0.0006 (11)
C9	0.0234 (13)	0.0292 (14)	0.0227 (13)	−0.0025 (11)	0.0101 (11)	−0.0005 (11)
C10	0.0244 (13)	0.0231 (13)	0.0178 (12)	0.0008 (10)	0.0110 (10)	0.0038 (10)
C11	0.0307 (15)	0.0270 (14)	0.0261 (14)	0.0025 (11)	0.0078 (12)	−0.0007 (11)
C12	0.0392 (16)	0.0280 (15)	0.0337 (15)	0.0072 (13)	0.0141 (13)	−0.0019 (12)
C13	0.0281 (14)	0.0364 (15)	0.0283 (14)	0.0096 (12)	0.0141 (12)	0.0089 (12)
C14	0.0236 (14)	0.0354 (15)	0.0255 (13)	0.0004 (11)	0.0093 (11)	0.0008 (12)
C15	0.0267 (14)	0.0265 (13)	0.0253 (13)	0.0019 (11)	0.0119 (11)	0.0012 (11)
N2	0.0232 (11)	0.0297 (12)	0.0201 (11)	0.0006 (9)	0.0084 (9)	−0.0007 (10)
O1	0.0474 (12)	0.0247 (10)	0.0324 (10)	0.0009 (9)	0.0149 (9)	−0.0043 (8)
O2	0.0330 (10)	0.0415 (11)	0.0188 (9)	0.0023 (8)	0.0093 (8)	0.0017 (8)
N1'	0.0249 (11)	0.0259 (11)	0.0214 (11)	0.0018 (9)	0.0057 (9)	0.0058 (9)
C2'	0.0284 (14)	0.0247 (13)	0.0220 (13)	0.0012 (11)	0.0107 (11)	−0.0031 (11)
O2'	0.0319 (10)	0.0242 (10)	0.0318 (10)	−0.0025 (8)	0.0093 (8)	0.0024 (8)
C3A	0.0217 (13)	0.0231 (13)	0.0204 (12)	0.0022 (10)	0.0083 (10)	−0.0025 (10)
C4'	0.0262 (14)	0.0244 (13)	0.0240 (13)	0.0048 (11)	0.0090 (11)	0.0017 (11)
C5'	0.0278 (14)	0.0259 (14)	0.0298 (14)	−0.0018 (11)	0.0131 (12)	−0.0023 (11)
C6'	0.0205 (13)	0.0325 (15)	0.0267 (14)	−0.0033 (11)	0.0082 (11)	−0.0093 (11)
C7'	0.0266 (14)	0.0293 (14)	0.0190 (12)	0.0033 (11)	0.0071 (11)	−0.0006 (11)
C7A	0.0276 (14)	0.0232 (13)	0.0200 (13)	0.0012 (11)	0.0102 (11)	−0.0019 (10)

### Geometric parameters (Å, °)

**Table:**

N1—C2	1.461 (3)	C10—C11	1.388 (3)
N1—C8	1.486 (3)	C11—C12	1.379 (3)
N1—C5	1.490 (3)	C11—H11A	0.9300
C2—C3A	1.530 (3)	C12—C13	1.372 (3)
C2—C2'	1.550 (3)	C12—H12A	0.9300
C2—C3	1.571 (3)	C13—C14	1.379 (3)
C3—N2	1.517 (3)	C13—H13A	0.9300
C3—C9	1.522 (3)	C14—C15	1.383 (3)
C3—C4	1.550 (3)	C14—H14A	0.9300
C4—C10	1.521 (3)	C15—H15A	0.9300
C4—C5	1.546 (3)	N2—O1	1.213 (2)
C4—H4A	0.9800	N2—O2	1.235 (2)
C5—C6	1.525 (3)	N1'—C2'	1.348 (3)
C5—H5A	0.9800	N1'—C7A	1.404 (3)
C6—C7	1.528 (3)	N1'—H1'	0.8544
C6—H6A	0.9700	C2'—O2'	1.224 (3)
C6—H6B	0.9700	C3A—C4'	1.383 (3)
C7—C8	1.521 (3)	C3A—C7A	1.387 (3)
C7—H7A	0.9700	C4'—C5'	1.389 (3)
C7—H7B	0.9700	C4'—H4D	0.9300
C8—H8A	0.9700	C5'—C6'	1.377 (3)
C8—H8B	0.9700	C5'—H5D	0.9300
C9—H9A	0.9600	C6'—C7'	1.384 (3)
C9—H9B	0.9600	C6'—H6D	0.9300
C9—H9C	0.9600	C7'—C7A	1.381 (3)
C10—C15	1.388 (3)	C7'—H7D	0.9300
			
C2—N1—C8	118.04 (18)	C3—C9—H9C	109.5
C2—N1—C5	109.64 (18)	H9A—C9—H9C	109.5
C8—N1—C5	108.79 (17)	H9B—C9—H9C	109.5
N1—C2—C3A	119.15 (19)	C15—C10—C11	117.7 (2)
N1—C2—C2'	108.24 (18)	C15—C10—C4	122.6 (2)
C3A—C2—C2'	101.33 (18)	C11—C10—C4	119.4 (2)
N1—C2—C3	99.68 (17)	C12—C11—C10	120.8 (2)
C3A—C2—C3	113.59 (18)	C12—C11—H11A	119.6
C2'—C2—C3	115.5 (2)	C10—C11—H11A	119.6
N2—C3—C9	106.34 (19)	C13—C12—C11	120.7 (2)
N2—C3—C4	113.56 (19)	C13—C12—H12A	119.6
C9—C3—C4	112.82 (19)	C11—C12—H12A	119.6
N2—C3—C2	106.86 (17)	C12—C13—C14	119.4 (2)
C9—C3—C2	115.85 (19)	C12—C13—H13A	120.3
C4—C3—C2	101.46 (18)	C14—C13—H13A	120.3
C10—C4—C5	114.60 (18)	C15—C14—C13	119.9 (2)
C10—C4—C3	119.50 (19)	C15—C14—H14A	120.1
C5—C4—C3	100.59 (18)	C13—C14—H14A	120.1
C10—C4—H4A	107.1	C14—C15—C10	121.3 (2)
C5—C4—H4A	107.1	C14—C15—H15A	119.4
C3—C4—H4A	107.1	C10—C15—H15A	119.4
N1—C5—C6	105.17 (18)	O1—N2—O2	124.0 (2)
N1—C5—C4	106.01 (17)	O1—N2—C3	120.35 (19)
C6—C5—C4	119.5 (2)	O2—N2—C3	115.60 (19)
N1—C5—H5A	108.6	C2'—N1'—C7A	111.4 (2)
C6—C5—H5A	108.6	C2'—N1'—H1'	123.1
C4—C5—H5A	108.6	C7A—N1'—H1'	124.5
C5—C6—C7	101.31 (19)	O2'—C2'—N1'	126.3 (2)
C5—C6—H6A	111.5	O2'—C2'—C2	124.9 (2)
C7—C6—H6A	111.5	N1'—C2'—C2	108.6 (2)
C5—C6—H6B	111.5	C4'—C3A—C7A	118.7 (2)
C7—C6—H6B	111.5	C4'—C3A—C2	133.3 (2)
H6A—C6—H6B	109.3	C7A—C3A—C2	108.1 (2)
C8—C7—C6	102.72 (19)	C3A—C4'—C5'	119.6 (2)
C8—C7—H7A	111.2	C3A—C4'—H4D	120.2
C6—C7—H7A	111.2	C5'—C4'—H4D	120.2
C8—C7—H7B	111.2	C6'—C5'—C4'	120.4 (2)
C6—C7—H7B	111.2	C6'—C5'—H5D	119.8
H7A—C7—H7B	109.1	C4'—C5'—H5D	119.8
N1—C8—C7	103.52 (18)	C5'—C6'—C7'	121.1 (2)
N1—C8—H8A	111.1	C5'—C6'—H6D	119.4
C7—C8—H8A	111.1	C7'—C6'—H6D	119.4
N1—C8—H8B	111.1	C7A—C7'—C6'	117.6 (2)
C7—C8—H8B	111.1	C7A—C7'—H7D	121.2
H8A—C8—H8B	109.0	C6'—C7'—H7D	121.2
C3—C9—H9A	109.5	C7'—C7A—C3A	122.6 (2)
C3—C9—H9B	109.5	C7'—C7A—N1'	126.9 (2)
H9A—C9—H9B	109.5	C3A—C7A—N1'	110.5 (2)
			
C8—N1—C2—C3A	−34.1 (3)	C4—C10—C11—C12	−172.1 (2)
C5—N1—C2—C3A	91.2 (2)	C10—C11—C12—C13	1.9 (4)
C8—N1—C2—C2'	80.8 (2)	C11—C12—C13—C14	−3.0 (4)
C5—N1—C2—C2'	−153.89 (18)	C12—C13—C14—C15	0.7 (4)
C8—N1—C2—C3	−158.11 (19)	C13—C14—C15—C10	2.9 (4)
C5—N1—C2—C3	−32.8 (2)	C11—C10—C15—C14	−3.9 (3)
N1—C2—C3—N2	165.61 (17)	C4—C10—C15—C14	169.5 (2)
C3A—C2—C3—N2	37.8 (2)	C9—C3—N2—O1	119.1 (2)
C2'—C2—C3—N2	−78.7 (2)	C4—C3—N2—O1	−5.6 (3)
N1—C2—C3—C9	−76.1 (2)	C2—C3—N2—O1	−116.6 (2)
C3A—C2—C3—C9	156.02 (19)	C9—C3—N2—O2	−61.1 (2)
C2'—C2—C3—C9	39.5 (3)	C4—C3—N2—O2	174.21 (19)
N1—C2—C3—C4	46.4 (2)	C2—C3—N2—O2	63.2 (2)
C3A—C2—C3—C4	−81.4 (2)	C7A—N1'—C2'—O2'	−174.3 (2)
C2'—C2—C3—C4	162.10 (19)	C7A—N1'—C2'—C2	1.2 (3)
N2—C3—C4—C10	77.2 (3)	N1—C2—C2'—O2'	47.5 (3)
C9—C3—C4—C10	−43.9 (3)	C3A—C2—C2'—O2'	173.6 (2)
C2—C3—C4—C10	−168.54 (19)	C3—C2—C2'—O2'	−63.2 (3)
N2—C3—C4—C5	−156.50 (18)	N1—C2—C2'—N1'	−128.0 (2)
C9—C3—C4—C5	82.4 (2)	C3A—C2—C2'—N1'	−1.9 (2)
C2—C3—C4—C5	−42.2 (2)	C3—C2—C2'—N1'	121.3 (2)
C2—N1—C5—C6	−120.7 (2)	N1—C2—C3A—C4'	−59.9 (4)
C8—N1—C5—C6	9.7 (2)	C2'—C2—C3A—C4'	−178.4 (3)
C2—N1—C5—C4	6.8 (2)	C3—C2—C3A—C4'	57.1 (3)
C8—N1—C5—C4	137.19 (19)	N1—C2—C3A—C7A	120.6 (2)
C10—C4—C5—N1	152.38 (19)	C2'—C2—C3A—C7A	2.1 (2)
C3—C4—C5—N1	22.8 (2)	C3—C2—C3A—C7A	−122.5 (2)
C10—C4—C5—C6	−89.2 (3)	C7A—C3A—C4'—C5'	1.6 (3)
C3—C4—C5—C6	141.2 (2)	C2—C3A—C4'—C5'	−177.9 (2)
N1—C5—C6—C7	−32.2 (2)	C3A—C4'—C5'—C6'	−0.7 (4)
C4—C5—C6—C7	−151.0 (2)	C4'—C5'—C6'—C7'	−0.7 (4)
C5—C6—C7—C8	42.7 (2)	C5'—C6'—C7'—C7A	1.3 (4)
C2—N1—C8—C7	142.8 (2)	C6'—C7'—C7A—C3A	−0.4 (4)
C5—N1—C8—C7	17.1 (2)	C6'—C7'—C7A—N1'	179.8 (2)
C6—C7—C8—N1	−37.2 (2)	C4'—C3A—C7A—C7'	−1.0 (4)
C5—C4—C10—C15	−27.0 (3)	C2—C3A—C7A—C7'	178.6 (2)
C3—C4—C10—C15	92.4 (3)	C4'—C3A—C7A—N1'	178.8 (2)
C5—C4—C10—C11	146.3 (2)	C2—C3A—C7A—N1'	−1.5 (3)
C3—C4—C10—C11	−94.2 (3)	C2'—N1'—C7A—C7'	−179.9 (2)
C15—C10—C11—C12	1.5 (4)	C2'—N1'—C7A—C3A	0.2 (3)

### Hydrogen-bond geometry (Å, °)

**Table:**

*D*—H···*A*	*D*—H	H···*A*	*D*···*A*	*D*—H···*A*
N1'—H1'···N1^i^	0.85	2.21	2.992 (3)	151

Symmetry codes: (i) *x*−1/2, −*y*+1/2, *z*−1/2.

## References

[bb1] Bruker (2007). *SMART* and *SAINT* Bruker AXS, Madison, Wisconsin, USA.

[bb2] Daly, J. W., Spande, T. W., Whittaker, N., Highet, R. J., Feigl, D., Noshimori, N., Tokuyama, T. & Meyers, C. W. (1986). *J. Nat. Prod.* **46**, 210–**???** 10.1021/np50044a012 3734811

[bb3] Grigg, R. & Sridharan, V. (1993). *Advances in Cycloaddition*, edited by D. P. Curran, Vol. 3, p. 161. London: Jai.

[bb4] Padwa, A. (1984). *1,3-Dipolar Cycloaddition Chemistry, *Vols. 1 and 2. New York: Wiley.

[bb5] Sheldrick, G. M. (1996). *SADABS* University of Göttingen, Germany.

[bb6] Sheldrick, G. M. (2008). *Acta Cryst.* A**64**, 112–122. 10.1107/S0108767307043930 18156677

[bb7] Usha, G., Selvanayagam, S., Velmurugan, D., Ravikumar, K. & Poornachandran, M. (2005). *Acta Cryst.* E**61**, o3312–o3314.

[bb8] Usha, G., Selvanayagam, S., Velmurugan, D., Ravikumar, K. & Raghunathan, R. (2005). *Acta Cryst.* E**61**, o3299–o3301.

[bb9] Waldmann, H. (1995). *Synlett*, pp. 133–141.

